# Creatinine clearance in selection of living kidney donor among the Malaysian population: is it safe?

**DOI:** 10.1186/s12882-023-03057-w

**Published:** 2023-09-11

**Authors:** Chee Keong Thye, Yee Wan Lee, Maisarah Jalalonmuhali, Soo Kun Lim, Kok Peng Ng

**Affiliations:** Division of Nephrology, Department of Medicine, University of Malaya, Jln Profesor Diraja Ungku Aziz, Kuala Lumpur, 59100 Selangor Malaysia

**Keywords:** Kidney transplant, Donor renal assessment, Creatinine clearance, Chromium 51 ethylenediamine-tetraacetic acid

## Abstract

**Background:**

Assessment of donor renal function is made by the measurement of Glomerular Filtration Rate (GFR). Exogenous markers are preferred over creatinine clearance and are widely used for measuring GFR. However, they are difficult to obtain, costly and laborious. This is a study to look into the safety and accuracy of creatinine clearance for renal assessment among the living kidney donors in the Malaysian population.

**Methods:**

This is a retrospective, single-centre study comprising 105 living kidney donor candidates from the year 2007 to 2020. By comparing against 51-Chromium ethylenediamine-tetraacetic acid (^51^Cr-EDTA), we analysed creatinine clearance for correlation, bias, precision and accuracy.

**Results:**

The study group had a mean age of 45.68 ± 10.97 years with a mean serum creatinine of 64.43 ± 17.68 µmol/L and a urine volume of 2.06 ± 0.83 L. Mean measured GFR from ^51^Cr-EDTA was 124.37 ± 26.83 ml/min/1.73m^2^ whereas mean creatinine clearance was 132.35 ± 38.18 ml/min/1.73m^2^. Creatinine clearance overestimated ^51^Cr-EDTA significantly with a correlation coefficient of 0.48 (*p* < 0.001) and an accuracy of 78.10% and 64.0% within 30% and 20% respectively of ^51^Cr-EDTA.

**Conclusion:**

Creatinine clearance is an acceptable and affordable alternative for donor renal assessment in the absence of exogenous markers with an emphasis on adequate urine collection followed by using measured GFR in selected cases.

## Background

From 2007 to 2016, there were a total of 1130 renal transplants in Malaysia, of which 426 were living-related renal transplants [1]. All living kidney donors undergo a series of workups prior to transplants and this includes assessment of renal function in the form of Glomerular Filtration Rate (GFR). An accurate assessment of GFR is important to minimize risks to the potential living kidney donor.

The gold standard for GFR assessment is by the measurement of urinary clearance of inulin. Other alternative exogenous markers include Chromium 51 ethylenediamine-tetraacetic acid (^51^Cr-EDTA), iothalamate, and iohexol. Of these, ^51^Cr-EDTA is well-recognized and one of the most widely used marker in the measurement of GFR [2]. However, these substances are difficult to obtain in Malaysia and their use are hampered by cost factors and laboriousness. Measuring creatinine clearance (CrCl) by means of 24-hour urine collection is an alternative when exogenous filtration markers are not available [3]. However, CrCl may be affected by overestimation or underestimation due to errors in urine collection as well as from the tubular secretion of creatinine [3–8].

There have been studies comparing various methods in measured GFR (mGFR) among potential living kidney donors and most have demonstrated superiority of using exogenous markers in comparison to creatinine clearance [4]. Nevertheless, there is a considerable variation of GFR evaluation among the transplant centres across the world [9, 10]. However, there is a lack of local data on the donor renal assessment in our country. Thus, this is a study to look into the safety and accuracy of CrCl against ^51^Cr-EDTA in measuring GFR among the living donors in Malaysian population in regards to feasibility as a first line agent.

## Methods

This is a retrospective, single-centre study. We looked into all adults aged 18 years and above who were potential living kidney donors from the year 2007 to 2020 with both measured GFR using ^51^Cr-EDTA and CrCl performed at the University of Malaya Medical Centre (UMMC).

A total of 180 living related adult kidney donors that underwent workups between the year 2004 and 2020 were recruited into the study. Each of these patients has up to 3 consecutive paired serum and urine creatinine samples and a ^51^Cr-EDTA measurement. Exclusion criteria include absence of serum/urine creatinine, absence of ^51^Cr-EDTA and inadequate 24-hour urine collection. For a 24-hour urine sample to be considered adequate, a general rule of urine creatinine ranging 177 to 221 µmol/kg/day for male and 133 to 177 µmol/kg/day for female was implemented across all ages [11]. 75 subjects (41.7%) were excluded due to inadequate urine sample using this criterion. Data collected from Electronic Medical Record (EMR) also included age, gender, race, weight, height, and Body mass index (BMI). Laboratory values include serum creatinine, 24-hour urine creatinine, and volume of urine.

Following a single intravenous administration of ^51^Cr-EDTA, blood was sampled at 2, 2.5, 3 and 4 h. Calculation of mGFR was performed using the slope-intercept method. Serum creatinine was determined by isotope dilution mass spectrometry reference-modified Jaffe kinetic assay (Cr_Jaffe_). Creatinine clearance (CrCl) was calculated using the formula:

$$\text{C}\text{r}\text{C}\text{l}(ml/\text{m}\text{i}\text{n})=\frac{{U}_{Cr}(mmol/24hr)\times Volume(ml/24hr)}{{S}_{Cr}(mmol/hr)\times 1440(min/24hr)}$$

where U_Cr_ represents urine creatinine and S_Cr_ represents serum creatinine. The resulting GFR from both ^51^Cr-EDTA and CrCl were adjusted to a body surface area of 1.73m^2^. BSA is determined using Du Bois Method [12]:

$$\text{B}\text{S}\text{A}=0.007184\times {\text{h}\text{e}\text{i}\text{g}\text{h}\text{t}}^{0.725}\times {\text{w}\text{e}\text{i}\text{g}\text{h}\text{t}}^{0.425}$$

Clinical data was statistically analysed using Statistical Package for the Social Sciences (SPSS) software (version 25.0; SPSS Inc., Chicago, IL, USA). A *p*-value of less than 0.05 is considered statistically significant. The data are shown as mean ± standard deviation (SD). To make a comparison between ^51^Cr-EDTA and CrCl, the correlation coefficient (*r*) was determined. The bias, precision, and accuracy within 30% and 20% of ^51^Cr-EDTA were also established. Bias was defined as the mean difference between CrCl and ^51^Cr-EDTA while precision of the CrCl is expressed as standard deviation of the mean difference between CrCl and ^51^Cr-EDTA. Accuracy between CrCl and ^51^Cr-EDTA incorporates both bias and precision and was expressed as percentage of CrCl falling within 30% and 20% respectively of ^51^Cr-EDTA.

The Bland-Altman plot were used as a graphical depiction of the above, where the bias was charted against the mean of the two methods. In another different approach, a modified Bland-Altman plot where ^51^Cr-EDTA, which is considered as a reference method, was portrayed on the *x* axis rather than using mean of ^51^Cr-EDTA and CrCl. The diagram is an alternative approach when one of the methods is considered more accurate [13].

## Results

Table 1 shows the baseline characteristics. The study group had a mean age of 45.68 ± 10.97 years with a mean serum creatinine of 64.43 ± 17.68 µmol/L and a urine volume of 2.06 ± 0.83 L. Female comprised 72.4% of the donors while Chinese, Malay and Indian made up 63.8%, 21.9% and 9.5% of the donors respectively. Mean mGFR from ^51^Cr-EDTA was 124.37 ± 26.83 ml/min/1.73m^2^ whereas mean CrCl was 132.35 ± 38.18 ml/min/1.73m^2^.

**Table 1:** Baseline characteristics

Characteristic (*n* = 105)	Mean ± SD or *n* (%)
Gender	Female 72 (72.4%)
Mean Age (years)	45.68 ± 10.97
Median Age (years)	45.00
Race	Chinese 67 (63.8%) Malay 23 (21.9%) Indian 10 (9.5%) Others 5 (4.8%)
BMI (kg/m^2^)	24.31 ± 3.97
BSA (m^2^)	1.64 ± 0.18
Plasma creatinine (µmol/l)	64.43 ± 17.68
Urine volume (L)	2.06 ± 0.83
Mean ^51^Cr-EDTA (ml/min/1.73 m^2^)	124.37 ± 26.83
Median ^51^Cr-EDTA (ml/min/1.73 m^2^)	122.00
Mean CrCl (ml/min/1.73 m^2^)	132.35 ± 38.18
Median CrCl (ml/min/1.73 m^2^)	122.91

**Fig. 1:**
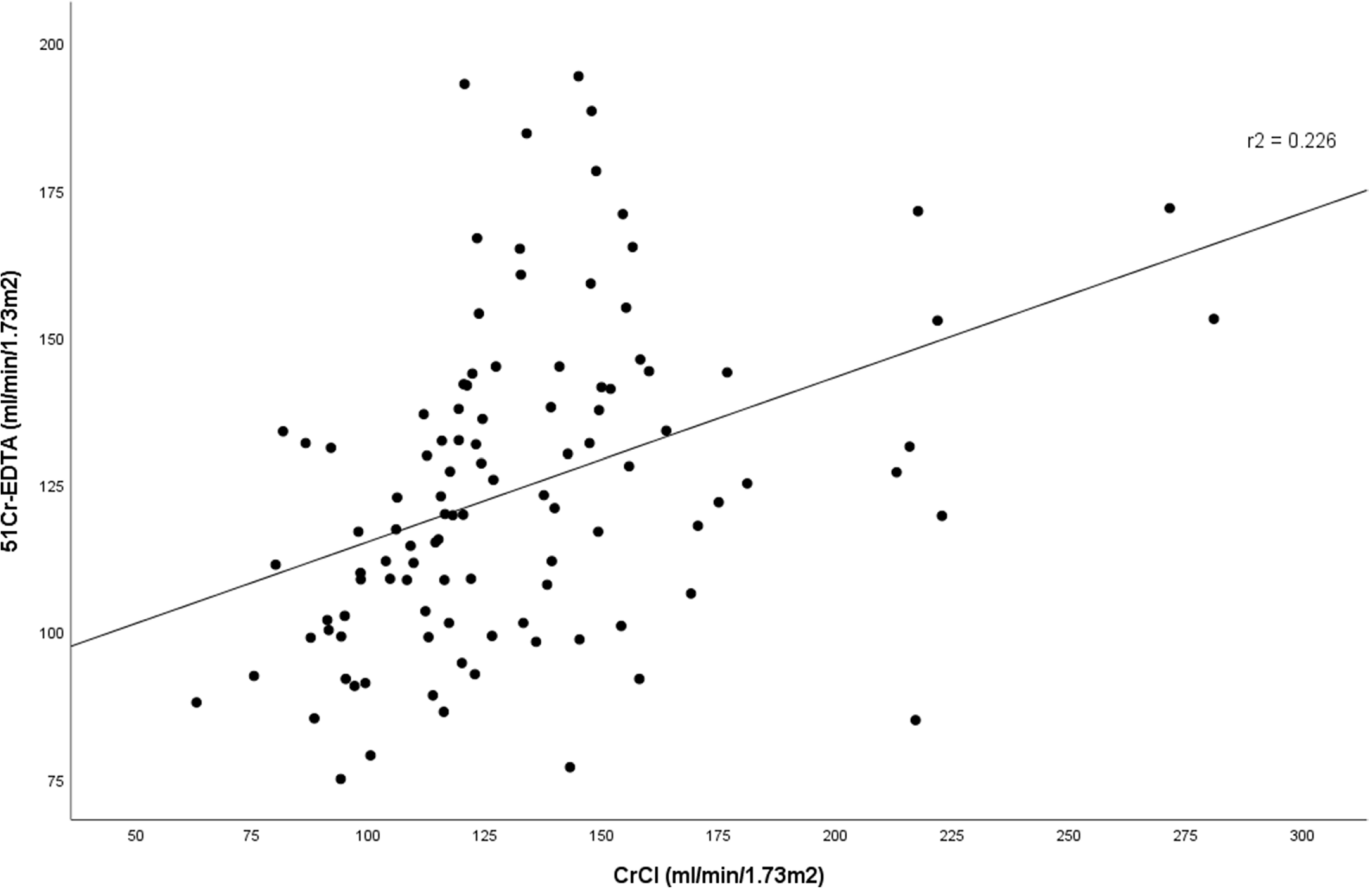
Relationship between ^51^Cr-EDTA and CrCl. The correlation coefficient is 0.48

The mean absolute bias between CrCl and ^51^Cr-EDTA was 7.98 ml/min/1.73m^2^ (8.7%) with a correlation coefficient (*r*) of 0.48. CrCl significantly overestimated ^51^Cr-EDTA (*p* < 0.001) (Fig. 1). This finding did not come as surprise as creatinine clearance is known to overestimate mGFR. However, the precision shown by CrCl in our study was suboptimal at 36.95 ml/min/1.73m^2^. The accuracy of CrCl within 30% of ^51^Cr-EDTA was 78.10%, but it dropped slightly to 61.0% when the accuracy within 20% of ^51^Cr-EDTA was used.

**Fig. 2:**
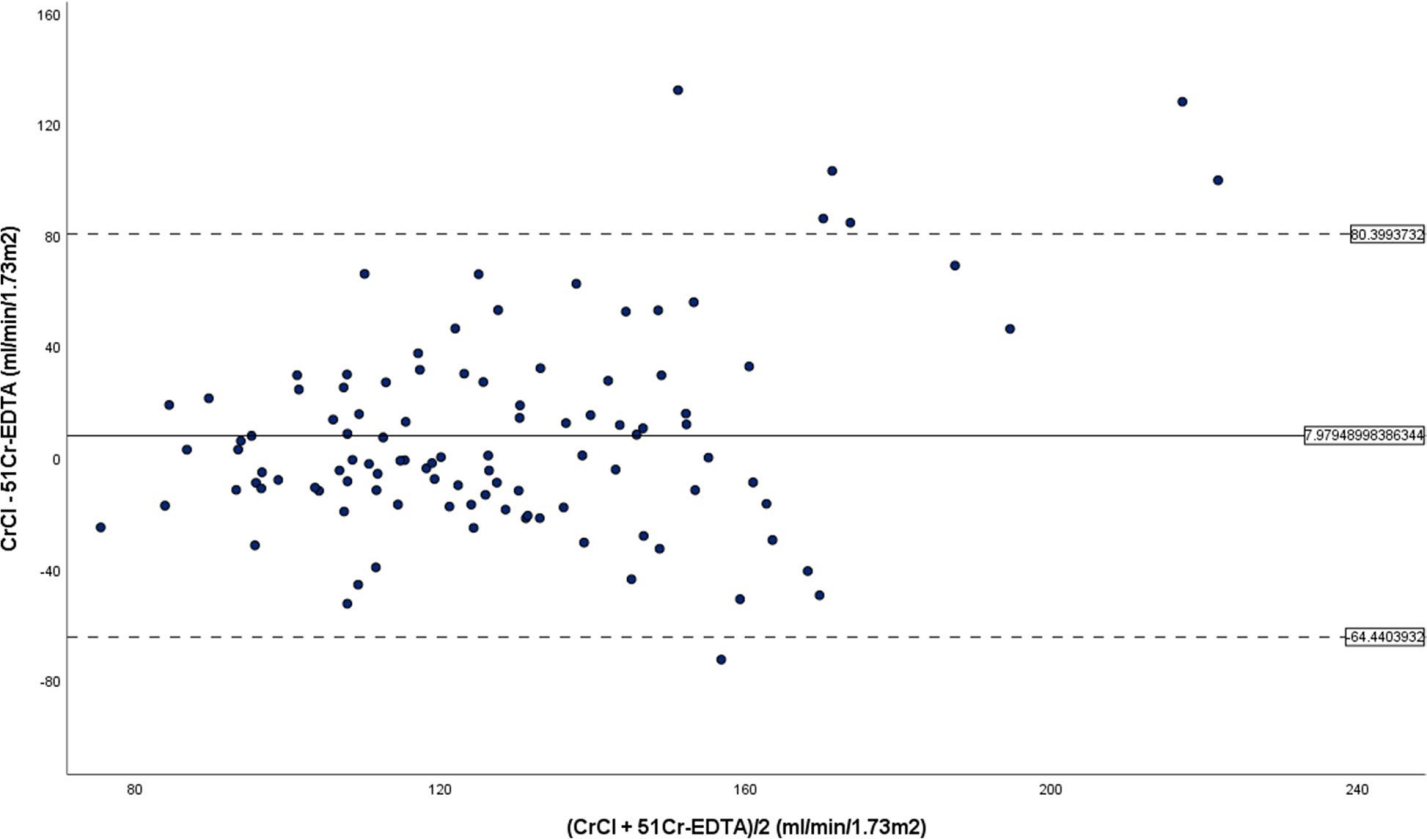
Bland and Altman plot whereby the differences are plotted against the mean between CrCl and ^51^Cr-EDTA. The solid line represents the mean difference (bias) while the dashed lines indicate ± 1.96 standard deviations (SD)

**Fig. 3:**
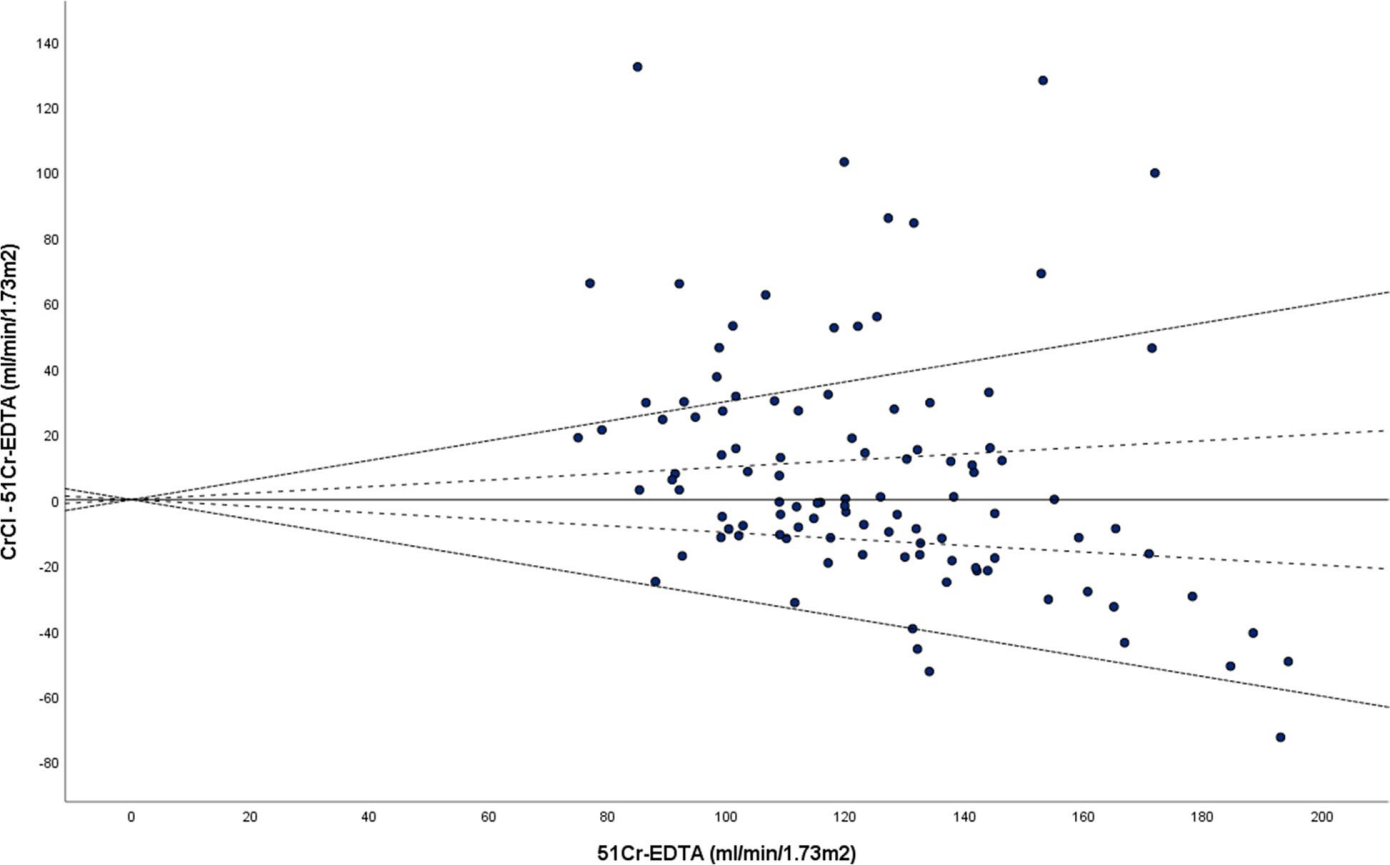
Modified Bland-Altman plot whereby the differences between CrCl and ^51^Cr-EDTA are plotted against ^51^Cr-EDTA (reference method) to visualize bias and accuracy. Accuracy within 30% (P_30_)(dotted lines) and 20% (P_20_)(dashed lines) of ^51^Cr-EDTA are shown

To further illustrate the difference between ^51^Cr-EDTA and CrCl, the Bland and Altman plot was used. This is a scatter plot (Fig. 2) that displays the span between − 2SD and + 2SD of the mean difference (limit of agreement that represents 95% confidence interval). Using the modified Bland and Altman plot (Fig. 3), we are able to appreciate that when CrCl is at a much higher reading than ^51^Cr-EDTA (beyond the threshold for kidney transplant) it is less accurate. The same cannot be said when CrCl is lower than its counterpart.

## Discussion

It is vital for donor renal assessment to be determined as accurately as possible. Malaysia has an incidence rate of renal transplantation of 3 to 5 per million population, which is very low in comparison to countries like Australia and New Zealand with a rate 27 to 37 per million population [1]. As Malaysia aims for a higher rate of living-related renal transplant, a precise assessment of GFR minimizes the long-term risks to the donors post-transplant. While Ibrahim et al. found that the survival and risk of end-stage kidney disease (ESKD) in a carefully screened donors are similar to the general population, in a longer follow up study done by Mjoen et al., they observed that kidney donors have a higher risk of cardiovascular deaths, ESKD and all-cause mortality [14, 15].

Clearance of chromium 51-labeled ethylenediaminetetraacetic acid (^51^Cr-EDTA) remains one of the most commonly used exogenous markers as an indirect measurement of GFR. Despite being more obtainable than inulin, ^51^Cr-EDTA remains scarce in Malaysia and it is also costly and technically more difficult to perform compared to CrCl. ^51^Cr-EDTA however, has a good accuracy of mGFR and this is shown by a systematic review of mGFR that compared 14 studies of ^51^Cr-EDTA against the reference method [4].

An mGFR of less than 80 ml/min/1.73m^2^ is excluded from kidney donation, which is important for donor outcome [16]. 24-hour urine collection for creatinine clearance remains the most common method for GFR assessment when the exogenous markers are not widely available. However, CrCl is fraught with reports of over and underestimation of GFR. KDIGO reports a magnitude of overestimation of 15% or more at normal GFR, based on older data using non-standardized serum creatinine assays [3]. Soveri et al., in their systematic review of mGFR found that CrCl overestimated renal inulin clearance of which 16 studies were of high quality [4].

Our study findings showed that CrCl had a significantly higher mean than using ^51^Cr-EDTA; which is in agreement with other similar studies that compared CrCl to the reference method [4, 5, 7, 8]. There were several plausible explanations for the overestimation. The most well-known circumstance would be the tubular secretion of creatinine, especially at higher readings of CrCl often encountered in healthy donors, which was also reflected on our data. Another recognised factor may come from inaccuracies in urine collection. We took into consideration on the adequacy of urine collection by gender and body weight to minimize sampling errors [11]. In this study, the adequacy was defined as urinary excretion of creatinine between 177 and 221 µmol/kg/day for male and 133 to 177 µmol/kg/day for female for all ages. However, it is also known that beyond 50 years of age, these figures progressively declines and thus in older candidates, there might be an overcollection leading to overestimation of mGFR.

Undeniably, by implementing the criteria of adequacy, only 58.3% of the donors had urine samples that were sufficient. The large proportion of donors with inadequate urine samples may reflect the lack of patient understanding of the urine collection procedure. Other reasons include loss of specimen from a poorly sealed container and incorrect storage at room temperature [17]. Indeed, our findings of inaccuracy were similar to the quoted rate of about 50% in other literatures. McGuire and colleagues found that 51% of patients had inaccurate urine collection while Sawyer and associates reported inaccurate urine sampling in 50.7% of their patients [18, 19].

To date, this is the first head to head study comparing the CrCl against ^51^Cr-EDTA. Despite the above shortcomings, it is important to note that CrCl in our study remained significantly concordant with ^51^Cr-EDTA with a reasonable accuracy within 20% and 30% of ^51^Cr-EDTA. By the above principles, it is probably acceptable in terms of utilizing CrCl for other purposes e.g. adjustments of dosage of medications. However, mGFR using CrCl for donor selection may have different implications as there exists a cutoff point for mGFR to be considered for donation. For example, if a potential kidney donor has a mGFR of 80 ml/min/1.73m^2^ using CrCl, it may be possible that the actual GFR may be lower. Reassuringly, from our study this is only observed at much higher levels of CrCl that is more than sufficient to be eligible for donation.

Even so, to address the overestimation of GFR by CrCl, we could potentially look into including urea and creatinine clearance in a single 24-hour collection [20]. We could also look into using cimetidine to improve the reliability of CrCl [21]. There were other limitations as well. Firstly, this is a retrospective single centre study with a small sample size. However, the study cohort has a multiracial composition which is in line with the multiethnicity in Malaysia. The inclusion of adequacy of urine collection also proved to have both favourable and unfavourable consequences as a large proportion of donors with inadequate urine samples were excluded from the analysis so as not to affect the validity of the study as well as the issue of possible overestimation in older donor candidates.

## Conclusion

The results of our study highlighted that CrCl is an acceptable and affordable alternative for donor GFR assessment with an emphasis on adequate urine collection. Taking into account on the overestimation bias, precision and accuracy, it is recommended for those with a CrCl below 100 ml/min/1.73m^2^ to proceed with mGFR using exogenous markers.

## Data Availability

The data that support the findings of this study are available on request from the corresponding author, KPN. The data are not publicly available as they contain information that could compromise the privacy of the participants.

## Abbreviations

^51^Cr-EDTA: 51-labeled ethylenediaminetetraacetic acid
BSA: Body Surface Area
BMI: Body Mass Index
CrCl: Creatinine clearance
ESKD: End Stage Kidney Disease
GFR: Glomerular Filtration Rate
mGFR: Measured Glomerular Filtration Rate
S_Cr_: Serum Creatinine
SD: Standard Deviation
SPSS: Statistical Package for the Social Sciences
U_Cr_: Urine Creatinine
